# Ileal Neuroendocrine Neoplasm: Diagnostic Imaging, Risk Factors, and Management Implications

**DOI:** 10.1002/ccr3.72620

**Published:** 2026-04-26

**Authors:** Mahfujul Z. Haque, Abdulmalik Saleem, Ammad Chaudhary, Taher Jamali, Kimberly Tosch

**Affiliations:** ^1^ Michigan State University College of Human Medicine Grand Rapids Michigan USA; ^2^ Department of Radiology Corewell Health Dearborn Michigan USA; ^3^ Gastroenterology Henry Ford Health System Detroit Michigan USA

**Keywords:** endoscopic, metastatic, neoplasm, neuroendocrine, small bowel, tumor

## Abstract

Early detection of small bowel neuroendocrine tumors (NETs), even when incidentally discovered, is crucial for improving outcomes. This case highlights the importance of recognizing incidental findings during imaging, as timely surgical intervention and multidisciplinary management can prevent progression, mitigate morbidity, and improve survival in these typically indolent but potentially metastatic tumors.

## 1 Introduction

Small bowel tumors (SBTs) are rare indolent tumors, comprising less than 0.6% of all new cancer cases in the United States [1]. Ileal neuroendocrine tumors (NETs) are well‐differentiated, SBTs composed of enterochromaffin cells [1]. Despite their indolent nature, NETs have a reported 10‐year survival of 65.5% [2]. Around 50% of small bowel NETs present with hepatic metastasis at the time of diagnosis, which can increase 5‐year mortality by 10%–20% [1, 3]. However, metastatic NETs often expedites diagnosis due to symptom manifestation.

Risk factors for the development of small bowel NETs include a history of smoking, chronic inflammatory conditions, and genetic predispositions. Given their indolence and high mortality risk, early detection is imperative. This case image details an asymptomatic ileal NET incidentally discovered during imaging for unrelated renal cysts in a patient with few risk factors.

## 2 Case History/Examination

We present the case of an asymptomatic 68‐year‐old African American male with a history of chronic kidney disease and tobacco use who underwent MRI of the abdomen for evaluation of renal cysts.

## 3 Differential Diagnosis, Investigations, and Treatment

**FIGURE 1:**
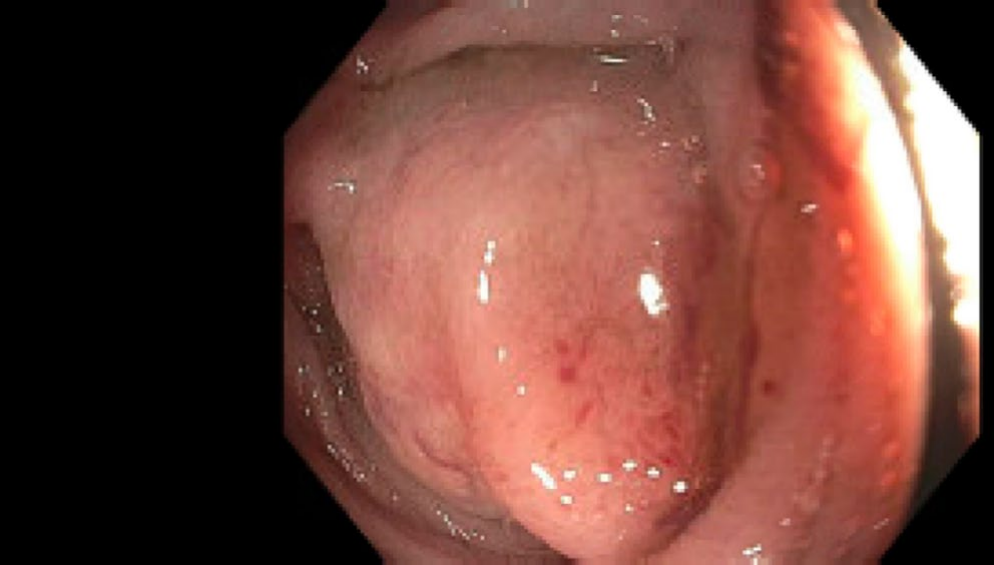
Endoscopic imaging of the partially obstructing tumor in the terminal ileum.

Computed‐tomography (CT) imaging demonstrated complex renal cysts in addition to an incidental finding of a non‐fatty enhancement of the ileocecal valve measuring 36 mm. DOTAPET scan demonstrated increased uptake in the cecum corresponding to the lesion along with non‐specific uptake in adjacent ileocecal lymph nodes with no distant metastatic disease noted. Subsequent colonoscopy identified a partially obstructing tumor spanning more than half the intestinal lumen in the terminal ileum, and it was concluded that this was the primary neoplasm (Figure 1).

## 4 Conclusion and Results (Outcome and Follow‐Up)

**FIGURE 2:**
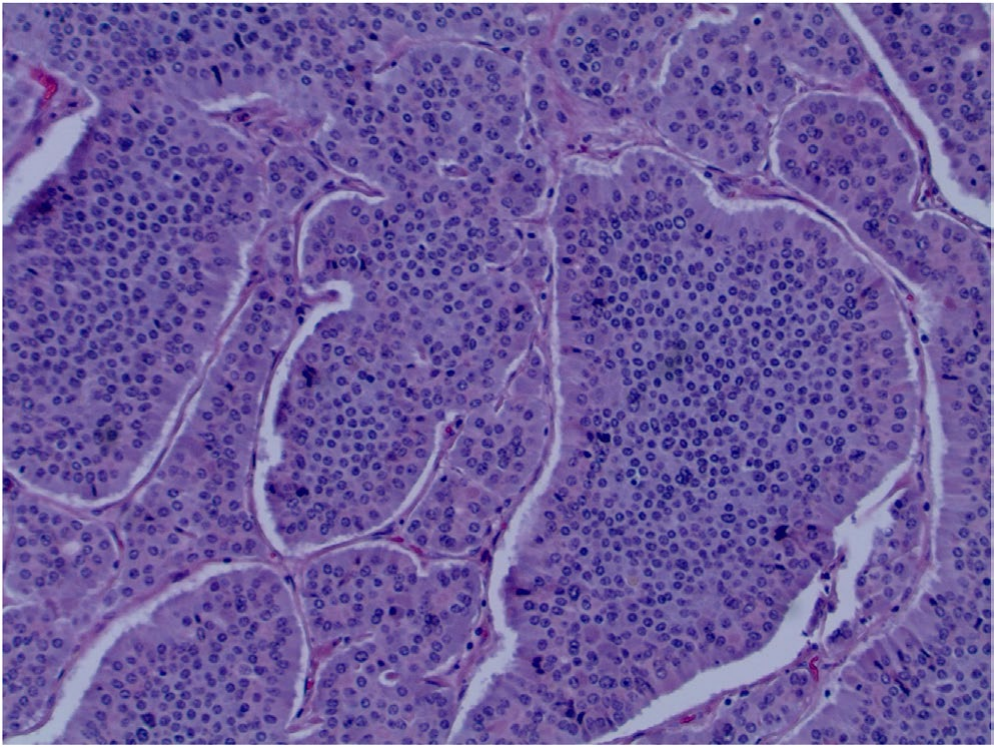
Hematoxylin and Eosin stain of the well‐differentiated neuroendocrine tumor.

The mass was biopsied, and pathology revealed a grade 1, well‐differentiated NET with tumor cells positive for Cam 5.2, synaptophysin, and Ki67 < 3% (Figure 2). A laparoscopic right hemicolectomy with small bowel resection was performed. Four out of thirteen excised adjacent lymph nodes were positive for metastatic NET. Our patient's postoperative course was complicated by ileus with eventual resolution.

## 5 Discussion

Data from the Surveillance, Epidemiology, and End Results registry demonstrates a consistent rise in the incidence of NETs from 1975 to 2012, with the starkest increase reported in individuals aged 40–69 years [3]. Ileal NETs, derived from enterochromaffin cells, are typically slow growing but possess the capacity to metastasize to distant organs, particularly the liver, which can drastically affect survival outcomes.

The tumor in our case was detected incidentally; despite occupying more than half of the intestinal lumen, the patient remained asymptomatic, an unusual presentation for a partially obstructing lesion of its size and location. A distinguishing feature of this case is the absence of hepatic metastasis despite lymph node involvement. Nearly 90% of patients diagnosed with GI‐NETs exhibit hepatic metastases at some point. While lymphatic spread was identified in four of thirteen examined nodes, the absence of distant metastasis highlights the benefits of early detection in altering disease progression favorably.

The patient was advised to return for CT imaging at 6‐month intervals and then transitioning to annual surveillance after the first year in the absence of recurrence, with consideration for cessation of surveillance after 10 years based on clinical progression. Therefore, clinicians should maintain a high index of suspicion for NETs, even in patients without a plethora of risk factors. Clinicians should recognize that NETS can be asymptomatic but have a favorable outcome when recognized, referred and appropriately managed.

## Author Contributions

**Mahfujul Z. Haque:** conceptualization, conceptualization, data curation, data curation, formal analysis, formal analysis, funding acquisition, funding acquisition, investigation, investigation, methodology, methodology, project administration, project administration, resources, resources, software, software, supervision, supervision, validation, validation, visualization, visualization, writing – original draft, writing – original draft, writing – review and editing, writing – review and editing. **Abdulmalik Saleem:** formal analysis, funding acquisition, investigation, investigation, methodology, methodology, project administration, project administration, software, software, supervision, supervision, validation, validation, visualization, visualization, writing – original draft, writing – original draft, writing – review and editing, writing – review and editing. **Ammad Chaudhary:** conceptualization, data curation, formal analysis, funding acquisition, investigation, methodology, project administration, supervision, writing – original draft, writing – review and editing. **Taher Jamali:** conceptualization, data curation, funding acquisition, project administration, software, supervision, writing – original draft, writing – review and editing. **Kimberly Tosch:** conceptualization, investigation, methodology, project administration, software, visualization, writing – original draft, writing – review and editing.

## Funding

The authors have nothing to report.

## Consent

Written informed consent was obtained from the patient to publish this report in accordance with the journal's patient consent policy.

## Conflicts of Interest

The authors declare no conflicts of interest.

## Data Availability

The data that support the findings of this study are available from the corresponding author upon reasonable request.
